# Unexpected Persistence of the Predatory Mite *Amblyseius andersoni* Under Insecticide Exposure in Italian Apple Orchards

**DOI:** 10.3390/insects17030338

**Published:** 2026-03-20

**Authors:** Guillaume Serra, Letizia Ripamonti, Venkata Avinash Addanki, Paola Tirello, Carlo Duso, Alberto Pozzebon

**Affiliations:** Department of Agronomy, Food, Natural Resources, Animals and Environment, University of Padua, Viale dell’Università 16, Legnaro, 35020 Padova, Italy; guillaume.serra@unipd.it (G.S.); letizia.ripamonti@studenti.unipd.it (L.R.); venkataavinash_addanki@sggw.edu.pl (V.A.A.); paola.tirello@unipd.it (P.T.); carlo.duso@unipd.it (C.D.)

**Keywords:** Phytoseiidae, predatory mites, biological control, pyrethroids, perennial cropping systems, integrated pest management

## Abstract

Predatory mites are key natural enemies in perennial crops, and their conservation is fundamental to ensure biocontrol of several pests. Yet their susceptibility to insecticides may compromise their role in Integrated Pest Management (IPM). In the past, pyrethroids proved to be poorly selective insecticides towards predatory mites, but in various countries their use recently increased in fruit orchards to counteract invasive pests. In this study, we assessed the effects of pyrethroids on the predatory mite *Amblyseius andersoni* through field experiments in apple orchards and laboratory assays on different strains. Field trials showed that *A. andersoni* populations remained unaffected by pyrethroid applications. Laboratory assays revealed intraspecific variation in *A. andersoni* populations: one commercial strain was highly susceptible to deltamethrin, whereas other commercial or field-collected strains were not affected, with no apparent sub-lethal effect on fecundity. These results underscore the contrasting susceptibility among predatory mite strains. From an IPM perspective, the lack of susceptibility to pyrethroids in *A. andersoni* may sustain biological control where insecticide use is unavoidable. Our findings stress the importance of evaluation procedures in toxicological studies, in particular the need to compare different strains, and of further investigation on predatory mite resistance. The implications for conservation programs in perennial cropping systems are addressed hereafter.

## Simple Summary

Predatory mites are minute arthropods that play an important role in agroecosystems by feeding on pest species. Because they provide natural pest regulation and help keep mite pests under economic injury levels, protecting and enhancing these natural enemies is important for sustainable crop production. However, non-selective insecticides used against insect pests may also affect these beneficial predators. In this study, we examined how pyrethroid insecticides affect predatory mite communities dominated by *Amblyseius andersoni* in apple orchards. We also tested in the laboratory whether various populations of the predatory mite *A. andersoni* differed in their susceptibility to these chemicals. Field results showed that a number of *A. andersoni* populations survived insecticide applications, in particular those based on pyrethroids, and maintained stable populations. Laboratory tests showed higher survival of *A. andersoni* collected in fruit orchards when exposed to deltamethrin than those from other sources. Implications for IPM are discussed.

## 1. Introduction

The conservation of biological control agents in agroecosystems is a key component of developing Integrated Pest Management (IPM) strategies to minimize the use of synthetic pesticides. However, non-selective pesticides are challenging the advances and effectiveness of IPM in agro-ecosystems. Specifically, the reduction in natural enemy populations due to the harmful effects of pesticides is a leading driver of secondary pest outbreaks, threatening the implementation of effective IPM strategies. Predatory mites are often considered non-target organisms, potentially affected by pesticides. These beneficial mites are known to struggle and lose their biocontrol activity because of repeated chemical pesticide application throughout the growing season [1]. Pesticides’ side effects on predatory mites can manifest in different ways, such as reduced survival, decreased reproduction, or altered behavior, ultimately decreasing their ability to suppress pest populations. Different studies showed that these lethal and sub-lethal effects can compromise the stability of natural enemy–pest interactions, leading to a weakened natural regulation and an increase in secondary pest outbreaks, e.g., [1,2,3,4,5]. Moreover, chemical stress on predatory mites may disrupt population dynamics and reduce the resilience of the entire agroecosystem, undermining IPM strategies. Therefore, evaluating the sensitivity of different predatory mite species and strains to commonly used insecticides is crucial for assessing potential risks, guiding sustainable chemical use, and conserving the long-term effectiveness of biocontrol agents.

Predatory mites are used in biological control programs due to their efficacy in suppressing populations of spider mites as well as of small insect pests such as thrips [6,7,8]. They are an important component of conservation biocontrol that relies on virtuous agricultural practices that naturally enhance the occurrence and density of beneficials through habitat management (e.g., agro-forestry, service/cover crops, flowering strips). In this context, the reduction in the adverse effects of pesticides is a prerequisite for any conservation practices [6,9,10,11]. Conservation biological control strategies deal in particular with generalist predatory mite species of the Phytoseiidae family. These species are particularly important, as not only do they prey on a broad range of pests but are also capable of persisting on plants in the absence of prey, feeding on alternative food sources such as pollen and fungi, including plant pathogens in some cases [6,8,12,13,14,15]. Generalist species can feed on a wide range of prey and alternative foods, maintaining their population even when prey populations are scarce, thus preventing herbivore outbreaks [16,17].

However, in agroecosystems, the persistence of predatory mite populations can be compromised by the use of non-selective pesticides [1,18]. This aspect is particularly concerning, as regulatory restrictions, particularly in Europe, continue to narrow the range of approved insecticides, thereby limiting the available options for pest management [19]. As a result, it is expected that future insecticides will rely on fewer chemical classes, and those based on pyrethroids, which generally have a favorable toxicological profile, are among the promising candidates. However, these compounds, acting as sodium channel modulators, targeting nerve and muscle functions (IRAC mode of action class 3A), are known to impact predatory mite populations negatively. Such effects pose a significant risk to the long-term success and sustainability of biological control programs [1]. In Europe, pyrethroids are intensively used to manage key and regulated pests (according to EU regulation 2031/2016) in fruit orchards, in particular against the invasive brown marmorated stink bug *Halyomorpha halys* (Stål) [20,21].

Pesticide toxicity on predatory mites has been widely studied over the past years [18,22,23,24,25,26,27,28] and some studies specifically showed reduced susceptibility or resistance to pyrethroids through field or laboratory forced selection in different species in Europe, Asia and the Americas: *Neoseiulus fallacis* Garman [29], *Metaseiulus occidentalis* (Nesbitt) [30,31], *Phytoseiulus macropilis* (Banks) [32], *Phytoseiulus persimilis* Athias-Henriot [33], *Amblyseius longispinosus* Evans [34], *Typhlodromus pyri* Scheuten, and *Amblyseius andersoni* (Chant) [35]. Both field and lab toxicological analysis are needed and lead to a better understanding of the effect on natural enemies [36,37,38]. Field studies can assess the effect in real-use scenarios, while laboratory studies are necessary to understand the effect at the individual level on life-history parameters, such as mortality and fecundity [2,3,4,38].

*Amblyseius andersoni* is one of the most important and widely distributed predatory mite species in European orchards, where it plays a key role in biological control programs [39,40,41,42]. However, the increasing pressure from invasive pests, specifically *Halyomorpha halys*, has led to a renewed reliance on broad-spectrum insecticides, including pyrethroids, in many European orchards. Although pyrethroids remain authorized and are widely used in Italy, their non-target effects on beneficial arthropods, including predatory mites, raise concerns about potential disruptions of biological control.

The aim of this study was to assess the effects of insecticide application programs on predatory mite populations in apple orchards. We focused on pyrethroid-based programs due to their predominant role in managing invasive pests. We first evaluated the impact of repeated pyrethroid applications on *A. andersoni* populations under field conditions. In this experiment, we used products sharing the same mode of action, but with different chemical traits that can potentially induce different effects on arthropods [43,44,45]. In the laboratory, we determined the effects of the pyrethroid deltamethrin on predatory mites’ survival and fecundity at the individual level. By integrating field-scale population responses with individual-level effects, this study provides a comprehensive assessment of the compatibility of pyrethroid use with conservation biological control in apple orchards.

## 2. Materials and Methods

### 2.1. Field Experiments

The effects of pyrethroid insecticides on predatory mites were investigated in three field experiments performed in apple orchards (Granny Smith (2022 and 2023), and Golden Delicious (2023)) located in the Veneto region, Northeastern Italy (Table 1). In all experiments, pyrethroid insecticides authorized for use in apple orchards were used at maximum recommended doses against the brown marmorated stink bug, *H. halys* (Table 1). In preliminary observations, orchards were colonized by naturally occurring predatory mites with *A. andersoni* as the only species, except for the Golden D. orchard in 2023, where *Kampimodromus aberrans* (Oudemans) was seldom observed. No spider mites were observed. Climatic conditions during experiments in the area were, in 2022, 27.2 ± 5.2 °C R.H. 65 ± 24%, and in 2023, 25.2 ± 6.5 °C R.H. 61 ± 25%. All experiments followed a randomized block design with four replicate plots per treatment of a minimum of 10 plants. The timing of applications was determined by local pest management practices outlined in the phytosanitary bulletin issued by the Regional Phytosanitary Service (https://www.regione.veneto.it/web/fitosanitario, accessed on 16 March 2026), and up to 6 applications were made in experimental apple orchards. Samplings were conducted before and 3 days after the first insecticide application and every 7 days until the 14th day after the last insecticide treatment (DAT). A total of 100 leaves per treatment (25 leaves per plot) were removed and transferred to the laboratory, where predatory mite individuals were counted under a dissecting microscope; a number of females were mounted on slides, in Hoyer’s medium, and identified under a phase contrast microscope using current identification keys.

Data were analyzed using generalized linear mixed repeated measures models with a lognormal error distribution and an identity link function, using the GLIMMIX procedure of SAS (v9.4). Mite densities were treated as response variables, with repeated measures taken at different sampling dates. Using an F test (α = 0.05), we evaluated the effect of insecticide application, time, and their interaction. Degrees of freedom were estimated using the Kenward–Roger method. According to Akaike’s Information Criterion, spatial power was chosen as the best-fitting covariance structure for correlating different sampling dates. The SLICE option of the LSMEANS statement was used to test treatment effect variation during observation periods, and contrasts (α = 0.05) were designed for pairwise comparison among treatments after insecticide applications. Untransformed data were used, and model assumptions were evaluated by inspecting diagnostic plots of model residuals. The effect (E) of insecticides was estimated using Henderson and Tilton’s formula.

### 2.2. Laboratory Experiments

This study was performed to evaluate the effect of the pyrethroid deltamethrin on five strains of *A. andersoni*, three of which were provided by different biocontrol companies referred to as C1 (CBC BioPlanet, Cesena, Italy), C2 (Biobest Group NV, Westerlo, Belgium), and C3 (Koppert Biological Systems, Berkel en Rodenrijs, The Netherlands), and two were collected from conventional and organic apple orchards in Northeastern Italy, respectively, FS-MAR (45°12′51.8″ N, 11°17′26.5″ E) and FS-DAL (45°21′06.6″ N, 11°12′51.5″ E). These populations were collected from orchards in the same area (Verona province) and managed similarly to the orchard where field experiments were performed. The conventional apple orchard is managed according to regional guidelines for voluntary IPM (Directive 2009/128/CE), which are defined each year by the Regional Phytosanitary Service, with diamides, neonicotinoids, and pyrethroids being the most frequently used insecticide groups. Insecticides used in organic apple orchards are regulated by Regulation (EU) 2018/848, with products based on *Bacillus thuringiensis*, natural pyrethrins, azadirachtin, and spinosad being the most commonly used. We tested multiple strains to assess potential intraspecific variability in susceptibility to this insecticide, as genetic and environmental differences among strains can influence tolerance to pesticides. Deltamethrin, a widely used type-II pyrethroid, was selected for laboratory bioassays because pyrethroids of this subclass are generally more toxic to arthropods than type I compounds [43,44,45]. Also, deltamethrin is the most frequently used pyrethroid, since, according to commercial product labels and regional guidelines for voluntary IPM, up to 4 applications per year are permitted, while a maximum of 2 is permitted for other insecticides in the same group. Experiments were conducted at a single concentration corresponding to the maximum recommended field dose to ensure ecologically relevant exposure conditions.

Predatory mites were reared in arenas consisting of a black plastic square placed on a wet sponge, with wet cotton layers as a barrier to prevent escape. Fresh pollen from *Typha* spp. (Nutrimite™, Biobest Group NV, Westerlo, Belgium) was provided three times a week. Rearing arenas were maintained in incubators at 25 ± 1 °C, 65 ± 10% RH and 16L:8D h photoperiod. All populations were reared for at least 2 months prior to experiments, corresponding to approximately 4 generations.

Experimental units consisted of a black plastic square (12 cm^2^) placed on wet cotton in a plastic box, with wet cotton barriers along the edges. Two 2-5-day-old females were transferred to each unit for a total of 40 females per treatment, divided into five replicates. After transferring the mites, experimental units were treated with a 2 mL of a 12,5 ppm deltamethrin solution, corresponding to the maximum field dose for Italian perennial crops (25 g/L, Decis^®^ Evo, Bayer CropScience S.r.l., Milano, Italy) or distilled water (as control), and sprayed at 6 bars using a Potter spray-tower (Burkard Scientific Ltd., Uxbridge, UK). Insecticide formulation was diluted in distilled water. After 30 min, when the residues were completely dried, pollen was provided to the mites. Experimental units were placed in an incubator at 25 ± 1 °C, 65 ± 10% RH, and 16L:8D h photoperiod.

Female survival was recorded at 72 h after insecticide exposure, while the fecundity of surviving females was recorded at 24, 72, and 168 h; eggs were removed to avoid cannibalism. Escaped and drowned females were withdrawn from the initial number for further analysis. Mites were considered dead if they did not move twice their size after being prodded with a fine brush. Fecundity was calculated as in [46]. Briefly, the number of eggs and the number of females present on the test units at each assessment date were used, taking into account a possible change in the number of females, following the formula of Blümel [47] with slight modifications. These corrected fecundity values were used to generate the graphs.

Data on survival were analyzed with R (version 4.2.3) using the function glm followed by a χ^2^ test (α= 0.05) to show the effects of factors. Differences among treatments were evaluated with a χ^2^ test of the least-squares means. Data on fecundity were analyzed with a factorial linear model using R (version 4.2.3), followed by an F-test (α= 0.05) to analyze the effect of the experimental factors, which were: *A. andersoni* strain, insecticide treatment and their interaction. Differences among treatments were evaluated with a *t*-test (α = 0.05) on the least-squared means. Before the statistical analysis, data on fecundity were checked for normality and homoscedasticity (both statistically and visually by plots) and were transformed using the package “rcompanion” in R (version 4.2.3), if necessary. All plots shown hereafter represent raw data.

## 3. Results

### 3.1. Field Experiments

#### 3.1.1. Experiment 1—Apple (Granny Smith, 2022)

**Figure 1:**
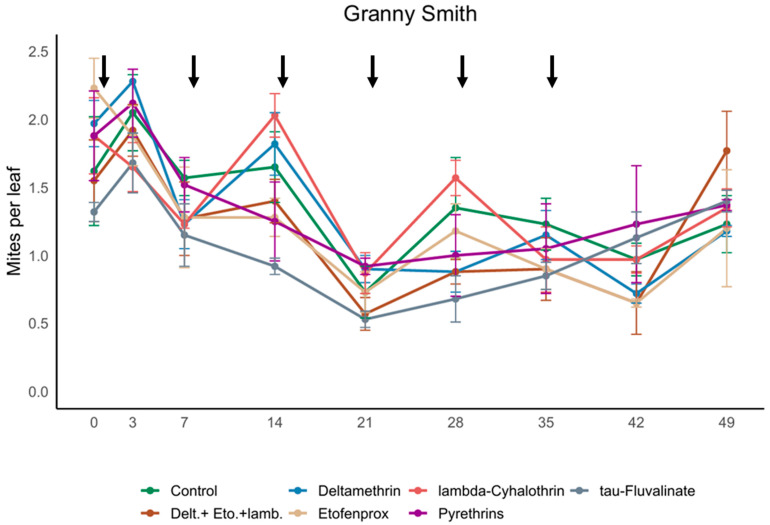
Average densities (mean ± std. err.) of *Amblyseius andersoni* observed in different treatments during the experiment performed in the apple orchard (2022). Arrows indicate the day of insecticide applications. On the X-axis, days after the first insecticide application are reported.

*Amblyseius andersoni* was observed as the dominant species in the experimental apple orchard. Its population density fluctuated during the experiment (F_8,101.9_ = 25.16; *p* < 0.001), but no effects of insecticides (F_6,14.51_ = 1.83; *p* = 0.163) and of the interaction between insecticide application and time (F_48,92.12_ = 1.26; *p* = 0.171) were observed (Figure 1).

#### 3.1.2. Experiment 2—Apple (Granny Smith, 2023)

**Figure 2:**
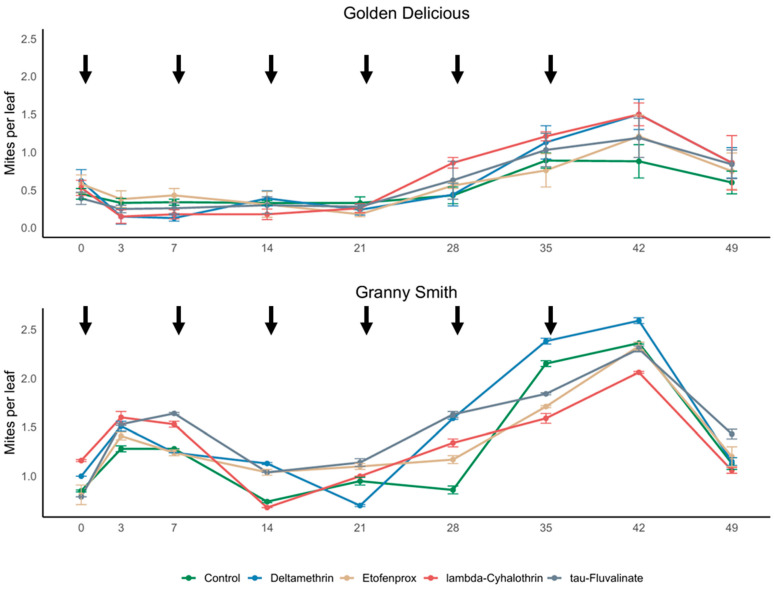
Average densities (mean ± std. err.) of *Amblyseius andersoni* observed in different treatments during the experiments performed in apple orchards (2023). Arrows indicate the day of insecticide applications. On the X-axis, days after the first insecticide application are reported.

The phytoseiid community in the apple orchard was represented only by *A. andersoni*. Its densities fluctuated during the experiment (F_8,120_ = 15.12; *p* < 0.001) while no effects of insecticides (F_4,15_ = 0.79; *p* = 0.551) and the interaction between insecticide application and time (F_32,15_ = 1.06; *p* = 0.399) were observed (Figure 2).

#### 3.1.3. Experiment 3—Apple (Golden Delicious, 2023)

*Amblyseius andersoni* was the dominant species also on this variety, and its numbers showed no significant variation during the experiment (F_2,43.29_ = 2.26; *p* = 0.116) and were not affected by insecticide applications (F_3,9.75_ = 0.02; *p* = 0.998) nor by the interaction between insecticide application and time (F_8,58.24_ = 0.67; *p* = 0.717; Figure 2).

### 3.2. Laboratory Experiments

#### 3.2.1. Lethal Effect of Deltamethrin

**Figure 3:**
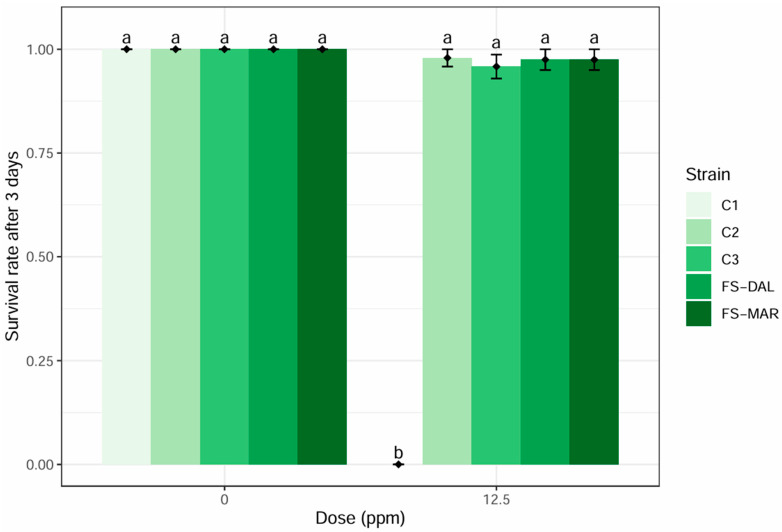
Effect of deltamethrin on the survival of five strains of *A. andersoni*. Different letters indicate significant differences by χ^2^ test (α = 0.05).

In the laboratory, a significant effect of deltamethrin treatment on different *A. andersoni* strains emerged (Table 2). Specifically, survival in the control was not different among the tested strains (Figure 3), while significant differences were observed among strains following field concentration (12.5 ppm) treatment with deltamethrin. The survival was 0% for the C1 strain, while survival rates were high for the other strains and the control, without significant differences among these treatments.

#### 3.2.2. Sub-Lethal Effects of Deltamethrin on Fecundity

**Figure 4:**
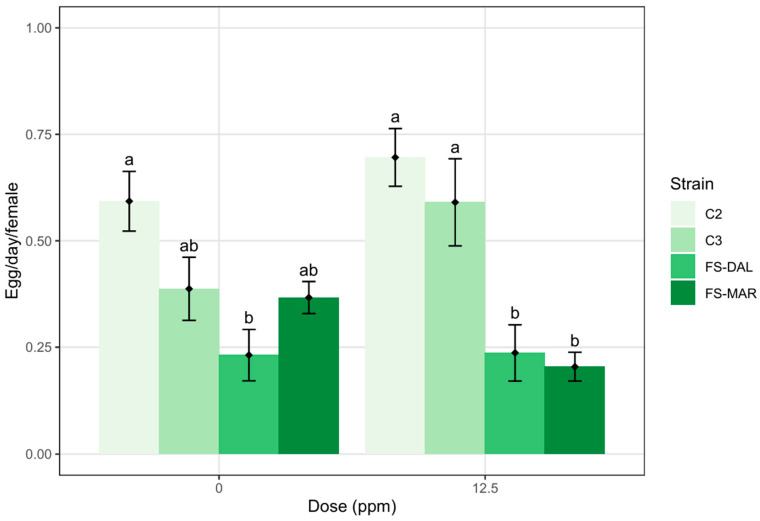
Effect of deltamethrin on fecundity of four *A. andersoni* strains, different letters indicate significant differences by a *t*-test (α = 0.05).

The fecundity of *A. andersoni* females was different among strains and was influenced by the strain × insecticide interaction, while no effect of deltamethrin emerged (Table 3). In the control treatment, the fecundity of the C2 strain was greater than that of FS-DAL, with C3 and FS-MAR showing intermediate levels. In deltamethrin treatment, C2 and C3 exhibited higher fecundity than the FS-DAL and FS-MAR strains (Figure 4).

## 4. Discussion

The results of our field experiments consistently indicate that *A. andersoni* populations were not significantly affected by the tested insecticide treatments, despite repeated pyrethroid applications. This finding was unexpected, as pyrethroids are generally considered non-selective and detrimental to predatory mites inhabiting apple orchards. However, our results showed the ability of apple-orchard populations of *A. andersoni* to persist under repeated pyrethroid exposure.

The dominance of *A. andersoni* in most apple-growing areas of north-eastern Italy has been highlighted in the past decades [39,48,49], and this situation was suggested to be influenced by their resistance to organophosphates [50]. This hypothesis was demonstrated for organophosphates and carbamates, while these strains proved to be susceptible to pyrethroids like deltamethrin [51,52,53]. In more recent studies, the impact of neonicotinoids was evaluated on *A. andersoni* populations occurring in the Trentino-Alto Adige region, and the reference toxic insecticide was the pyrethroid tau-fluvalinate. The latter insecticide proved to be detrimental to *A. andersoni,* confirming the non-selectivity of pyrethroids on this predatory mite [54]. Despite the detrimental effects of pyrethroids, *A. andersoni* was still dominant in northern Italian apple orchard communities. Among factors affecting this status, we can mention their resistance to widely used fungicides [55] and its competitiveness towards other heterospecific phytoseiids [56,57,58]. *Amblyseius andersoni* has also been found as a dominant species in other European apple-growing areas [40,59,60,61,62].

Our study adds novel evidence compared to previous work. Previous studies demonstrated pyrethroid resistance in vineyard-collected strains of *A. andersoni*, and in one case, an apple-collected strain was used as a susceptible reference [35,63]. In contrast, our results indicate that apple-orchard populations can also exhibit reduced susceptibility to pyrethroids. Moreover, Bonafos et al. [35] relied exclusively on laboratory bioassays, whereas our study integrates both field data and individual-level laboratory assessments. This combined approach strengthens the ecological relevance of our findings and supports the hypothesis that reduced susceptibility may contribute to the long-term persistence and dominance of *A. andersoni* in pyrethroid-treated apple orchards.

The harmfulness of pyrethroids for predatory mites belonging to the family Phytoseiid has been highlighted in the 1970s and the 1980s [64,65,66,67,68]. Therefore, these insecticides were excluded from IPM programs. The occurrence of *H. halys* in Italy was followed by severe damage to the fruit-growing areas of Northern Italy, and, as in other parts of the world, the invasion by this pest led to a strong increase in broad-spectrum pesticide use, with pyrethroids as the main products [20,69,70]. These insecticides are an important component of crop protection plans in several Italian regions despite the expectation of spider mite outbreaks. Surprisingly, this phenomenon was not widely observed, and this study can explain the main factor involved: *A. andersoni* populations can tolerate repeated applications of pyrethroids.

The laboratory experiments confirmed that field strains were not susceptible to deltamethrin and revealed distinct patterns of survival and fecundity among the *A. andersoni* strains, whether or not they were exposed to deltamethrin, underscoring divergent inter-strain responses after pesticide application. Therefore, the impact of pyrethroids on *A. andersoni* appeared to differ among the tested strains. The survival rate remained high for all strains after field-dose exposure to deltamethrin, while the C1 strain exhibited 100% mortality, showing a marked susceptibility compared to the other strains. Regarding fecundity, our results revealed differences among the strains considered. The strains showed different fecundity rates under deltamethrin exposure depending on their origins. While the company strain displayed the highest fecundity, the field-collected strains showed lower daily egg production. This suggests that the fitness of field strains may be lower than that of mass-reared mites and may depend on genetic background and environmental factors such as resource availability, predation, and prior pesticide exposure [71,72,73,74,75]. Fecundity within the same strain after exposure to deltamethrin was not significantly reduced, indicating no sub-lethal effect of the treatment in those strains. The absence of sub-lethal effects on fecundity observed in the present study was somewhat unexpected, as numerous studies have reported negative reproductive effects of pyrethroid exposure in phytoseiid mites. In a previous study on the effect of another pyrethroid (i.e., tau-fluvalinate) on *A. andersoni,* a reduction in survival and fecundity was observed [54]. Considering other predatory mite species, pyrethroids, and deltamethrin in particular, have frequently been associated with both lethal and sub-lethal effects on predatory mites, including reductions in fecundity and longevity. For instance, Zanuzo Zanardi et al. [76] reported high toxicity of pyrethroids to *Iphiseiodes zuluagai* Denmark & Muma, with effects exceeding those observed for the neonicotinoids tested. In that study, pyrethroid exposure significantly reduced both fecundity and longevity of adult females. Similarly, Savi et al. [77] found that deltamethrin caused high adult mortality in *Phytoseiulus longipes* Evans and induced pronounced sub-lethal effects on surviving individuals, including increased developmental time and reductions in fecundity, fertility, and longevity. Comparable patterns have been reported in other phytoseiid species. For example, Chang et al. [78] showed that pyrethroids exhibited the greatest toxicity among several insecticide classes tested—including macrocyclic lactones, neonicotinoids, carbamates, organophosphates, and organochlorines—with deltamethrin being the most toxic compound to *Neoseiulus agrestis* (Karg), with negative effect on its survival and fecundity. Chen et al. [79] ranked deltamethrin as more toxic to *Neoseiulus barkeri* (Hughes) than other tested insecticides, including chlorpyrifos, abamectin, and imidacloprid. More broadly, reductions in female oviposition following pyrethroid exposure have been reported in multiple phytoseiid species, including *Euseius gossipii* (El-Badry) [80], *Neoseiulus californicus* (McGregor) [81], *N. fallacis* [82], *P. persimilis* [83], and *Galendromus occidentalis* (Nesbitt) [3,84]. In contrast to these findings, no significant reduction in fecundity following deltamethrin exposure was detected in the strains tested in the present study. One possible explanation for this difference is that most previous studies evaluated susceptible predatory mite populations, whereas the strains examined here (excluding C1) were tolerant to deltamethrin with no effect on survival and fecundity. The mechanisms underlying the association between the absence of an effect on survival and reproduction remain unclear, and future research should address this.

Our findings demonstrate that deltamethrin did not induce sub-lethal effects on the fecundity of *A. andersoni* that survived deltamethrin treatment, whereas it caused a clear lethal effect in a strain that appears to be susceptible. This strain was not later tested for a sub-lethal effect on fecundity, as no females were found alive after the exposure to deltamethrin. The commercial strains also appeared to have higher fecundity in laboratory conditions than the field-collected ones, probably corresponding to selection pressure under mass-reared conditions for generations. While this might be interesting for pest control, those mites could have a much lower genetic diversity, causing a potential problem under other selective pressures like extreme temperature/humidity fluctuation under climate change conditions [85,86].

Strains exhibiting high survival after insecticide treatment could be considered candidates for biological control strategies in fields where chemical treatments cannot be avoided. The sharp contrast between the strains highlights that not all populations likely share the same tolerance background, reinforcing the importance of evaluating more strains per species in toxicological studies. Importantly, to our knowledge, this is the first study to jointly demonstrate field and strain-dependent tolerance to pyrethroids in *A. andersoni* populations.

Overall, our results emphasize the need for selective pesticide use and careful evaluation of their side effects on predatory mite communities. Future work should focus on the compatibility of different insecticides with natural enemy conservation, investigate resistance, clarifying its molecular and genetic basis within predatory mites, including potential target-site mutations and metabolic detoxification [33,87,88], to better understand how resistance is maintained and transferred to offspring.

## Figures and Tables

**Table 1:** Treatments, insecticides, doses and application timing applied in field experiments. Manufacturer: ^1^ Serbios S.r.l., Badia Polesine (RO), Italy; ^2^ Bayer CropScience S.r.l, Milano, Italy; ^3^ Adama Italia S.r.l., Grassobbio (BG), Italy; ^4^ Sipcam Italia S.p.A., Milano, Italy; ^5^ Syngenta Italia S.p.A., Milan, Italy.

Treatments—Active Ingredients	Commercial Products (a.i. Concentration and Manufacturer)	Dose (mL/hL)	Application Number and Timing (Day from First Application)
Experiment 1—Apple (cv. Granny Smith) 2022			
Control	-	-	-
Pyrethrins	Asset Five (5%) ^1^	64 mL/hL	6; day 0, 7, 14, 21, 28, 32
Deltamethrin	Decis^®^ Evo (2.42%) ^2^	60 mL/hL	6; day 0, 7, 14, 21, 28, 32
Tau-Fluvalinate	Mavrik^®^ Smart (21.4%) ^3^	120 mL/hL	6; day 0, 7, 14, 21, 28, 32
Etofenprox	Trebon^®^ Up (30%) ^4^	50 mL/hL	6; day 0, 7, 14, 21, 28, 32
Lambda-Cyhalothrin	Karate Zeon^®^ (9.48%) ^5^	30 mL/hL	6; day 0, 7, 14, 21, 28, 32
Deltamethrin + Etofenprox + Lambda-Cyhalothrin	Decis^®^ Evo (2.42%) ^2^	60 mL/hL	4; day 0, 7, 14, 21
	Trebon^®^ Up (30%) ^3^	50 mL/hL	1; day 28
	Karate Zeon^®^ (9.48%) ^5^	30 mL/hL	1; day 32
Experiment 2 and 3—Apple (cv. Granny Smith and Golden Delicious) 2023			
Control	-	-	-
Deltamethrin	Decis^®^ Evo (2.42%) ^2^	60 mL/hL	6; day 0, 7, 14, 21, 28, 32
Tau-Fluvalinate	Mavric^®^ Smart (21.4%) ^3^	120 mL/hL	6; day 0, 7, 14, 21, 28, 32
Etofenprox	Trebon^®^ Up (30%) ^4^	50 mL/hL	6; day 0, 7, 14, 21, 28, 32
Lambda-Cyhalothrin	Karate Zeon^®^ (9.48%) ^5^	30 mL/hL	6; day 0, 7, 14, 21, 28, 32

**Table 2:** Results of the GLM model analysis of variance on the survival of five *A. andersoni* strains under laboratory conditions after 3 days.

Survival			
	*df*	*Chi2*	*p*
Strain	4	9.52	<0.001
Insecticide	1	2.72	<0.001
Strain × Insecticide	4	25.41	<0.001

**Table 3:** Results of the linear model analysis of variance on fecundity of *A. andersoni* strains under laboratory conditions.

Fecundity			
	*df*	*F*	*p*
Strain	3	17.64	<0.001
Insecticide	1	0.054	0.816
Strain × Insecticide	3	3.078	0.029

## Data Availability

The original contributions presented in this study are included in the article. Further inquiries can be directed to the corresponding authors.
